# Healthy Communities Program

**Published:** 2008-12-15

**Authors:** Carlos Santos-Burgoa, Lucero Rodríguez-Cabrera, Elvia Macedo de la Concha, Evangelina Álvarez García, Adrenalina Cebrián Gómez

**Affiliations:** General Director of Health Promotion; Dirección General de Promoción de la Salud, Secretaría de Salud, Mexico.; Dirección General de Promoción de la Salud, Secretaría de Salud, Mexico.; Dirección General de Promoción de la Salud, Secretaría de Salud, Mexico.; Dirección General de Promoción de la Salud, Secretaría de Salud, Mexico.

## Introduction

Access to health services should be universal, and health promotion interventions must be designed with respect for the cultural, environmental, and socioeconomic characteristics of individual communities. The Healthy Communities Program (HCP) is a response to the need to democratize health. HCP generates local health promotion projects that are designed to improve quality of life through community participation with authorities from the federal to the local level.

HCP began within the framework of Mexico's National Health Program 2001-2006, the objectives of which were to improve the health of the Mexican population, reduce health disparities, guarantee quality care in public and private health care institutions, and strengthen the National Health System, particularly in the public sector, by using specific strategies and action plans (1).

HCP has since established itself as a priority initiative for creating or strengthening health promotion activities in all the nation's municipalities. It requires the participation of different sectors of the population — public, private, and community (here, community is loosely defined as a group of people with similar values who live in the same area; this definition includes community-based organizations and informal neighborhood groups [2]) — to conduct health education and prevention activities and promote healthy behaviors.

## Rationale for HCP

Mexico consists of 2,442 municipalities (equivalent to counties in the United States) distributed among 32 states; the municipality is the basic unit of political-administrative structure in Mexico and the level of government that is closest to the needs of the population. At the level of the municipality, citizens interact closely with government authorities to reach consensus, resolve conflicts, and work toward the public good; urban development, public services, waste recycling programs, culture and tourism services, and disaster preparedness all take place at the level of the municipality. This is also the level at which citizens and government institutions can work together most effectively to develop economically sustainable health policies. We believe that a healthy community can result only when municipal authorities, community-based institutions, and citizens come together in support of policies and programs that improve the health and quality of life of all community members and their families (2).

HCP's roots date back to the 1980s, when the World Health Organization promoted the Healthy Cities Initiative, first in developed countries and then, in the 1990s, in Latin American countries. As part of this initiative, the Mexican government implemented the Healthy Municipalities Program in 1995; for the first time, local authorities from the health sector encouraged citizens and community groups to work together to solve the principal health problems that affected their community and their quality of life. Results of the Healthy Municipalities Program were positive; communities perceived that working with local authorities was beneficial. Encouraged by these results, the federal government began to systemize health promotion activities and their implementation in a new structure, HCP.

## How HCP Works

HCP integrates successful local health promotion programs so that resources are used more effectively, efforts are not duplicated, and multiple partners can combine their resources. Integrating local programs increases awareness of health issues in the community, develops capacity, and improves the quality of life of community members. HCP principally operates in 2 broad areas: municipal participation in health promotion and community health organization.

### Municipal participation in health promotion

Municipal participation requires all authorities, not just those in health-related fields, to be involved with establishing a municipal health committee, in conjunction with community organizations and the private sector. This committee is in charge of assessing municipal health status, defining municipal health priorities, and establishing the municipal health promotion program. This program addresses health issues facing the community, such as availability of potable water, solid waste management, and pest control.

HCP solicits ideas for nationwide projects from municipal health committees. The HCP committee evaluates these projects according to 18 health themes: 1) environmental safety, 2) zoonosis control, 3) vectorborne disease, 4) solid waste management, 5) addiction prevention, 6) healthy communities, 7) accident prevention, 8) health promotion, 9) healthy markets and sanitary animal slaughter, 10) adult and older adult health, 11) basic sanitation, 12) dental health, 13) children's and teenagers' health, 14) reproductive health, 15) tuberculosis, 16) potable water, 17) HIV/AIDS prevention, and 18) others. HCP sponsors projects that meet all technical requirements. Municipalities can be certified by HCP and also receive funds from other programs, such as the Healthy Mexican Municipalities Network.

### Community health organization

Community health organization consists of engaging citizens in improving the health of community members and improving the environment by being members of the municipal health committee. Members of the committee are involved in implementing local health promotion activities.

## Goals of HCP

HCP has 5 basic imperatives: developing policies to improve public health, supporting municipal projects linked to public health, promoting health education and health communication, strengthening the capacity of the public health workforce, and self-monitoring and evaluation.

### Developing policies to improve public health

This strategy supports the implementation of the Municipal Health Promotion Program and develops technical and legislative mechanisms that facilitate program implementation.

### Support for municipal projects linked to public health

HCP provides technical and financial assistance to municipal projects that promote community health; these projects are collectively referred to as the Municipal Health Promotion Program. These projects implement new technologies or interventions that improve the health or socioeconomic condition of a community. Projects may deal with basic sanitation, the use of latrines in very poor communities, solid waste management, or reproductive health education for women (eg, a program about the benefits of screening with the Papanicolaou test).

### Health education and health communication

HCP designs and implements health communication and education campaigns aimed at increasing community members' health knowledge and awareness of healthy behaviors. Examples of past programs include "Health Starts at Home," "Women and Health," and "Basic Hygiene."

### Strengthening the capacity of the public health workforce

HCP assesses training needs and designs ad hoc training sessions for health promotion personnel (including doctors, nurses, social workers, midwives, health promoters, and members of the municipal health committee) at the state, municipal, and local level. Training sessions cover various health themes and strategies on how to implement public health interventions in the community. Sessions take place at the local levels; they involve classroom participation and use appropriate educational materials.

### Self-monitoring and evaluation

Since its inception, HCP has required the methodic, consistent, and accurate measurement of its components, such as sponsored projects, number of healthy communities, and number of people who attend programs. This measurement allows public health authorities to determine the effectiveness of HCP interventions at the state level so that programs can be improved and implementation problems can be identified and corrected. Monitoring the program also increases transparency in how its resources are managed and makes any limitations apparent.

## HCP Structure and Organization

Because HCP is a national program, it is under the jurisdiction of the Health Secretariat through the General Directorate of Health Promotion. The Health Secretariat defines the operational guidelines that states use to administer the program, stimulates and supports local planning, and supports operations and program monitoring. Health secretariats at the state level share responsibility with the states for program administration, through the state health promotion offices.

The sanitary jurisdiction is responsible for the organization, coordination, follow-up, and control at the municipal and local levels of HCP; the primary care units are responsible for providing health care services. Health promoters support health promotion, disease prevention, and health care at the community level. The local health committee is the primary unit of integration; at this level, community members, in coordination with health personnel, can improve environmental conditions in the community and receive health education.

Because of the nature of HCP, communities and the diverse institutions that are part of Mexico's health system must be engaged for the program's long-term objectives — creating health promotion activities and strengthening existing activities in all the nation's municipalities — to be realized. These objectives can only be achieved by assigning human and material resources to support functions of diverse technical complexity, in varied levels of responsibility.

## Conclusion

In 6 years of implementing HCP, we have learned that engaging local authorities and community members is essential. Participation of local authorities tends to decline, especially after local elections (every 3 years), and newly elected authorities must be lobbied to participate in HCP. At this point, community involvement is crucial because community members are most effectively positioned to engage the participation of their newly elected officials.

Municipal projects have been successful because they are not constrained by federal or state authorities. Health problems are identified at the local level, and solutions are implemented on site. For example, from 2002 to 2006, HCP sponsored 1,059 projects throughout Mexico (3); these projects involved 71% of all the municipalities. Some municipalities are poorly accessible, which presents obstacles to implementing programs in these areas.

Of the 18 health themes, solid waste management is the most common type of HCP-sponsored program (16.8%), followed by zoonosis control (12.5%) and vectorborne disease (9.2%). The total budget for these programs from 2001 through 2006 was 160,726,788 pesos (US $14,611,526).

Evaluating all the components of HCP has given us information to modify aspects of it that are not working. For example, communication and coordination strategies have had to be adjusted so that communication between federal and local authorities was most effective.
